# Epithelial Sodium Channel in the Respiratory System: A Bibliometric Review of Recent Studies

**DOI:** 10.3390/biology15110864

**Published:** 2026-05-30

**Authors:** Yunmei Fu, Yifan Yao, Hong-Long Ji, Hongguang Nie

**Affiliations:** 1 Department of Stem Cells and Regenerative Medicine, College of Basic Medical Science, China Medical University, Shenyang 110122, China; 2022110021@cmu.edu.cn; 2 Department of Pharmaceutical Toxicology, School of Pharmacy, China Medical University, Shenyang 110122, China; yaoyifan1@cmu.edu.cn; 3 Department of Surgery, Stritch School of Medicine, Loyola University Chicago, Maywood, IL 60153, USA; hji@luc.edu; 4 Department of Microbiology and Immunology, Stritch School of Medicine, Loyola University Chicago, Maywood, IL 60153, USA; 5 Burn and Shock Trauma Research Institute, Stritch School of Medicine, Loyola University Chicago, Maywood, IL 60153, USA

**Keywords:** bibliometric analysis, epithelial sodium channel, inflammatory responses, pathophysiology, acute lung injury

## Abstract

Inhibiting epithelial sodium channel (ENaC) provides clinical benefit in cystic fibrosis treatment, whereas activated alveolar ENaC attenuates acute lung injury/acute respiratory distress syndrome. However, seldom bibliometric analysis of respiratory ENaC systematically evaluates hotspots and trends in ion transport, as well as providing few overviews for researchers. This review aims to provide a comprehensive overview of respiratory ENaC over the past 40 years, identifying the research frontier and topic evolution. Searching the literature in Science Citation Index Expanded from Web of Science (WOS) and PubMed, a total of 1634 publications in 409 journals, authored by 5457 researchers from 60 countries/regions, were analyzed by CiteSpace, VOSviewer, and sciMAT software. The earliest highly cited article, titled ‘Rescue of CF airway epithelial cell function in vitro by a CFTR potentiator, VX-770’, had accumulated 1011 citations. The most prolific author, country, and journal were MA Mall, USA, and *Am J Physiol Lung Cell Mol Physiol*, respectively. In the most recent phase of annual focus, research areas could be clustered by immunology, cell biology, pharmacology and pharmacy, biotechnology and applied microbiology, and physiology. In summary, this bibliometric research reveals that respiratory ENaC is not only responsible for alveolar fluid clearance, but has converted into a broader modulator in immune-inflammatory responses.

## Simple Summary

The epithelial sodium channel (ENaC) plays an important role in maintaining fluid homeostasis and host defense in the respiratory system, while the overall research trends and key players in this field remain unclear. In this bibliometric review, we analyzed 1634 publications from 1988 to 2025 to map the evolution of respiratory ENaC research. Our findings reveal a clear shift from basic molecular studies toward pathophysiological mechanisms involving inflammation, alveolar fluid clearance, and immune regulation. The United States, Germany, and China are the most productive countries, with leading authors including Mall, Boucher, and Matthay. This review provides a comprehensive overview for researchers and highlights future directions, particularly the role of ENaC in inflammatory responses and fluid transport in pulmonary diseases. Understanding the trends of respiratory ENaC can help guide new therapeutic strategies and identify patients at risk.

## 1. Introduction

First identified in the late 20th century for its fundamental role in sodium absorption across epithelium, the epithelial sodium channel (ENaC) belongs to the ENaC/degenerin gene superfamily, which is widely distributed throughout multiple organs and species [1,2]. Structurally, ENaC in humans is composed of a heterotrimer with α, β, γ, and δ subunits, encoded by SCNN1A, SCNN1B, SCNN1G, and SCNN1D, respectively, which assemble to form a functional channel modulated by proteolytic cleavage, intracellular signaling pathways, and extracellular conditions [3,4,5]. Of note, the data from genomics identify that human SCNN1D is located between UBE2J2 and ACAP3 genes on chromosome 1p36, genetic deletion of which are predisposed to respiratory infection and the development of nasal congestion [6]. The alveolar epithelium controls hydration of the airway surface liquid by transepithelial ion transport-induced osmotic pressure, which drives the movement of water [7,8]. Functionally, ENaC plays a vital role in maintaining the fluid balance in the lungs and contributing to innate host defense mechanisms [9]. Within the respiratory tract, the activity of ENaC is finely tuned to maintain optimally hydrated airway surface liquid, which is vital for effective mucociliary clearance, a key innate defense mechanism [10].

In cystic fibrosis, the mutation of the cystic fibrosis conductance regulator (CFTR) leads to excessive ENaC activity, causing the depletion of airway surface liquid and defective mucus transport [11]. Conversely, α-ENaC-deficient mice with loss of neonatal lung liquid clearance develop respiratory distress and die within 40 h of birth [12]. In a similar, acute lung injury and its more severe progression, acute respiratory distress syndrome, are both life-threatening respiratory conditions, characterized by defective ENaC activity, leading to increased alveolar permeability, severe pulmonary edema, and hypoxic respiratory failure [13,14,15]. Considering its prominent role in the pathophysiology and omics study of the respiratory system, ENaC has become a promising therapeutic target for preclinical evaluation and treatment strategy [16]. In cardiovascular diseases, the solution of activated ENaC-induced vascular dysfunction and hypertension can be achieved by personalized precision medicine [17,18]. For respiratory diseases, ETD001, a novel inhaled ENaC blocker, exhibited a long active duration for people with cystic fibrosis in a Phase 2 clinical study [19].

During the past 15 years, significant progress in molecular and functional characterization of ENaC has been made, while the features in respiratory diseases are still under exploration [20]. Till now, bibliometric analysis has been widely applied in the field of medical research [21]. However, bibliometric studies on ENaC in the respiratory system have not been reported. This study aims to identify hotspots and trends in ion transport, which will provide an overview for researchers already involved or those who want to become involved in the field.

## 2. Materials and Methods

### 2.1. Data Sources and Search Strategies

**Figure 1:**
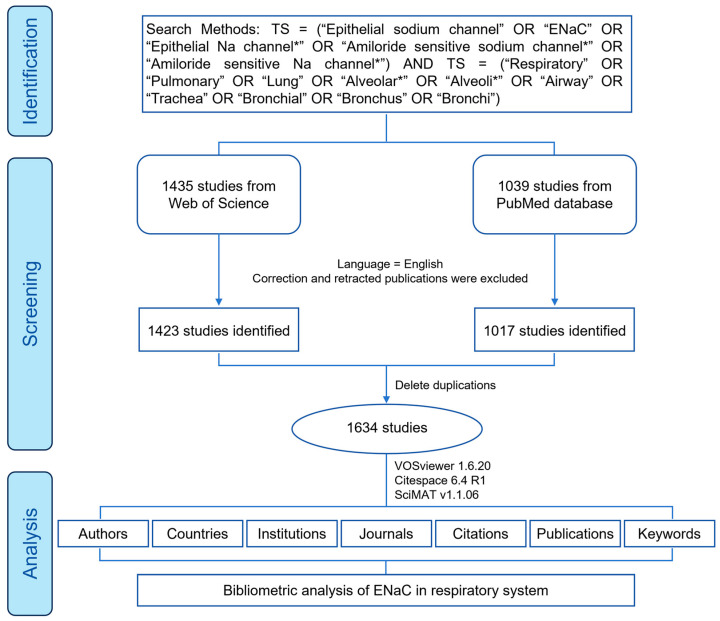
The overall flowchart of this bibliographic study on respiratory ENaC publications.

A systematic literature search was conducted in the Science Citation Index Expanded from Web of Science (WOS) and PubMed on 22 August 2025. All publications were downloaded on a single day to avoid changes in citation counts and records due to daily database updates. In order to explore the current situation and trends about ENaC in the respiratory system, the retrieval strategy was topic sentence (TS) = (“Epithelial sodium channel” OR “ENaC” OR “Epithelial Na channel*” OR “Amiloride sensitive sodium channel*” OR “Amiloride sensitive Na channel*”) AND TS = (“Respiratory” OR “Pulmonary” OR “Lung” OR “Alveolar*” OR “Alveoli*” OR “Airway” OR “Trachea” OR “Bronchial” OR “Bronchus” OR “Bronchi”). The data were collected from 1988 to 2025, the language chosen was English, and corrected and retracted publications were excluded. Two investigators (Y. Fu and Y. Yao) performed manual verification independently and resolved any discrepancies through discussion, resulting in the exclusion of duplicate records. All data were sourced from public databases and did not involve human participants, so ethical approval was not required. The detailed literature selection and screening process was illustrated in Figure 1.

### 2.2. Data Collection

After completing the literature selection from the WOS and PubMed databases, EndNote X9, a reference management software, was used to remove duplicates from both databases. A manual verification of all records was subsequently undertaken to scrutinize the data accuracy. A total of 810 duplications were excluded; the remaining 1634 publications were converted to .txt format and imported into CiteSpace 6.4 R1 (Philadelphia, PA, USA) for bibliometric analysis.

### 2.3. Statistical Analysis

Bibliometric analysis, firstly introduced in 1969, was a quantitative method used to analyze literature within a given research field, and helps identify the current focus and emerging trends by extracting key metrics. Combined with modern visualization tools, bibliometric results could be presented more intuitively and informatively. In this study, visualizations were generated using CiteSpace 6.4 R1 and VOSviewer (v1.6.20, Leiden, The Netherlands) to offer complementary features. CiteSpace was used to visualize publication trends, keyword bursts, and citation bursts [22]. Parameters were set as follows: time span (from 1988 to 2025, with a 1-year slice interval), g-index (k = 25), minimum duration (2 years), and burst parameters were kept at their default values. During the analyses of authors, countries, and keywords in the literature research, a cosine similarity threshold was used to reflect the proximity of academic direction by measuring angular differences within vector space. VOSviewer generated co-occurrence networks of authors, journals, institutions, and citations [23], and the information about documents, citations, and H-index was collected with the assistance of WOS.

Additionally, the evolution of research themes was analyzed using sciMAT (v1.1.06, Granada, Spain), which incorporated methods, algorithms, and measures for all the steps in the general science mapping workflow, from preprocessing to the visualization of the results [24]. Firstly, morphologically similar keywords were automatically and manually grouped, while irrelevant terms were excluded *via* stop groups. For example, irrelevant generic terms, including study, review, and analysis, were excluded using both the software’s built-in stop word list and manually added terms. Full names and their corresponding abbreviations were standardized, such as ENaC and epithelial sodium channel. Then the documents were divided into three phases (1988–1999, 2000–2015, and 2016–2025). The development of the themes was analyzed through the established thematic evolution, and clusters were positioned in a strategic diagram along centrality (external relevance) and density (internal development) axes, defining four thematic quadrants: driving and relevant themes (Q1), entrenched/isolated themes (Q2), emerging/declining themes (Q3), and cross-cutting or underdeveloped themes (Q4).

## 3. Trends of Publications

ENaC is the key channel protein for pulmonary edema fluid clearance, serving as the rate-limiting step for sodium and water reabsorption from the alveolar space into the circulation [2,25]. In this section, two visualizations and three tables were used to analyze the interactions, publications, citations, and H-index of authors, as well as countries, and journals regarding the field of respiratory ENaC.

### 3.1. Annual Publication

**Figure 2:**
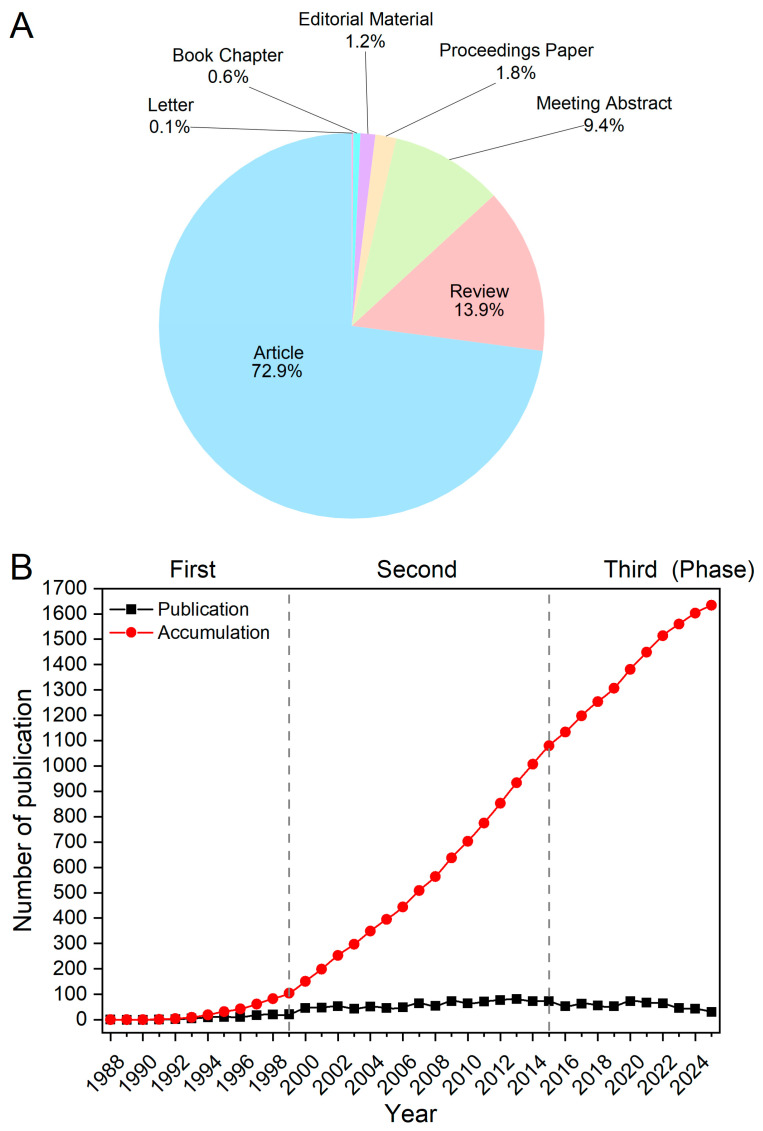
The document type and annual publication of ENaC in the respiratory system from 1988 to 2025. (**A**) Document type of publication, (**B**) the number of annual/cumulative publications.

The WOS and PubMed databases were used to search the literature related to ENaC and the respiratory system published from 1 January 1988 to 22 August 2025 by topic. For types of publications presented in Figure 2A, a total of 1634 studies were retrieved from the WOS and PubMed databases, including 1191 articles (72.9%), 227 reviews (13.9%), and 153 meeting abstracts (9.4%), as well as other documents (1.0%).

As shown in Figure 2B, the cumulative number of publications from 1988 to 2025 showed an upward trend, which could be divided into three phases based on publication output. In 1988, an amiloride-sensitive Na^+^ channel was first isolated from bovine trachea using an affinity gel and reconstituted into a planar lipid bilayer [1]. ENaC presented in the alveolar epithelial cell membrane was confirmed to affect net salt and water reabsorption, the dysregulation of which in cystic fibrosis and pulmonary edema was found to be opposite [26,27]. Accordingly, a significant increase in research output was witnessed, and the annual number of documents increased from 46 to 81 [28,29]. Of note, the development of transgenic mouse models, which induced airway-specific overexpression of ENaC or knockout of upstream protein, enabled in vivo investigation of ENaC function and strengthened the crucial role of ENaC in the treatment of respiratory diseases [30,31]. The publications of respiratory ENaC research continued to increase in the recent 10 years, possibly due to advanced techniques, such as single-particle cryo-electron microscopy and single-cell RNA sequencing [32,33]. Intriguingly, the number of publications suddenly increased during 2020–2021, a period that coincided with the outbreak of the COVID-19 pandemic [34,35]. ENaC dysregulation through inhibition of plasmin-dependent cleavage and activation of GPCR signaling pathways was one of the mechanisms underlying alveolar fluid imbalance in COVID-19 patients, demonstrating how a severe global health crisis could rapidly redirect research priorities within an established field [36,37,38].

### 3.2. Keywords Analysis

**Figure 3:**
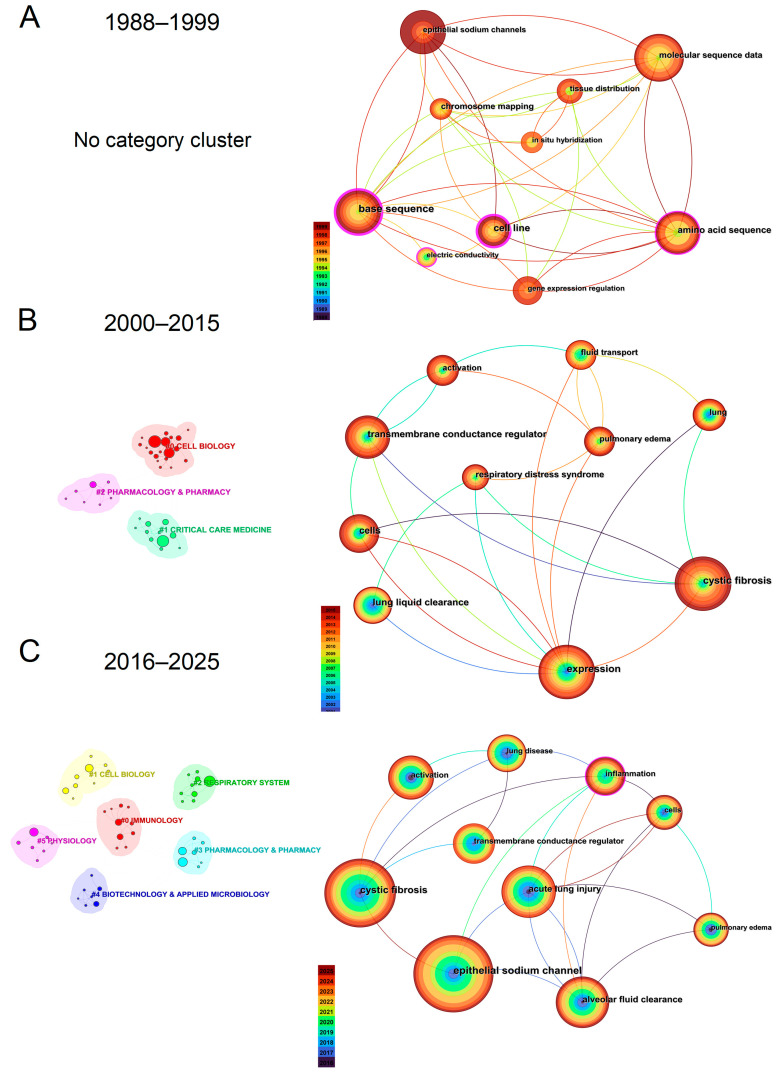
The analysis of keywords. The category clusters and keywords co-occurrence network map from (**A**) 1988–1999, (**B**) 2000–2015, and (**C**) 2016–2025. **Left**: category clusters, and the sequence numbers of the clusters are sorted by the size of the clusters. The smaller number (#) indicates the bigger cluster, and different colors represent different clusters. **Right** is the co-occurrence network map. The different colors of circles and lines represent the years studying in the ENaC of the respiratory system. The circle sizes represented the frequency of keywords, and the length of lines represented the strength of collaboration among authors.

The co-occurrence network analysis of keywords aimed to identify the research hotspots in a specific field during a certain period by studying the terms that summarized the content of the literature [21]. Based on the category clusters and keyword co-occurrence networks of three phases (Figure 3), the evolution of ENaC in the respiratory system could be systematically analyzed. In the first phase (1988–1999), due to the relatively small number of publications, there was no category cluster. The top ten frequently occurring keywords were mainly base sequence, amino acid sequence, and molecular sequence data, reflecting a foundational period focused on gene discovery and basic biophysical properties, and these keywords began to emerge mainly after 1996. In the second phase, there was a pathophysiological expansion about functional ENaC in cystic fibrosis, pulmonary edema, and fluid transport mechanisms, indicating a translational shift towards disease mechanisms. In the most recent phase (2016–2025), more categories were clustered, including immunology, cell biology, respiratory system, pharmacology and pharmacy, biotechnology and applied microbiology, and physiology. Compared with the second phase, we found immunology ranked first, which indicated that immune-related research has become a key hotspot in the respiratory ENaC at present. Additionally, category clusters of cell biology and pharmacology and pharmacy continued from the second stage to the third stage, and the cluster of biotechnology and applied microbiology emerged, signifying a critical and maturing status for the field under the development of advanced biotechnology. Keywords connecting ENaC function of inflammation, acute lung injury, and alveolar fluid clearance highlighted the role of Na^+^ transport in critical illness and immune-inflammatory pathways.

### 3.3. Highly Cited Papers

The top 10 most cited papers, including 6 reviews and 4 articles, further illustrated the central themes of ENaC in respiratory system research (Table 1). The journals named Physiol Rev and Annu Rev Physiol appeared twice, suggesting the review could provide guidance for subsequent research. However, the most cited reference, an article written by Van Goor, F in Proc Nat Acad Sci USA, was cited 1011 times and focused on VX-770, a CFTR potentiator, to reduce excessive Na^+^ and fluid absorption to prevent dehydration of the apical surface and increase cilia beating in epithelial cultures [39]. Notably, a substantial body of reviews and articles systematically investigated the regulation, function, and pathophysiology of respiratory ENaC, thereby highlighting the significant translational potential of ENaC.

Among these top 10 highest cited references, the most recent study was the review written by Ji, HL, who found that the activation of ENaC by plasmin-induced cleavage played an important role in COVID-19 susceptibility [35], indicating continued evolution and expanding research interest in ENaC, particularly in pandemic respiratory diseases.

## 4. General Information

In this section, two visualizations and three tables were used to analyze the interactions, publications, citations, and H-index of authors, countries, and journals regarding the field of ENaC in the respiratory system.

### 4.1. Ranking Author’s Productivity

Around 5457 authors contributed over 1634 publications involving respiratory ENaC. Then we ranked the top 10 most productive authors according to citations. All of these authors contributed a total of 459 publications, making for 28.09% of the total. Among them, 7 of the top 10 most productive authors were affiliated with institutions in the USA, while the remaining 3 authors were from Germany, Switzerland, and China, respectively (Table 2). The top three authors were Mall, MA (Charité-Universitätsmedizin, Berlin, 66 publications), Boucher, RC (University of North Carolina, 60 publications), and Matalon, S (University of Alabama at Birmingham, 56 publications). Among these 10 most productive authors, the top three authors with the highest citations were Boucher, RC, Mall, MA, and Matthay, MA. So, this phenomenon drove us to explore the main research directions of the above four authors. Matalon, S made extensive contributions to ENaC dysfunction caused by respiratory viral disease and cystic fibrosis [40,41]. At present, Mall, MA deeply focused on functional lung imaging to identify peripheral ventilation changes and lung functional improvement by mesenchymal stromal cells using the adult β-ENaC-transgenic mouse model [20,42]. Boucher, RC was an expert in cystic fibrosis, with research centering on the role of ENaC in airway epithelium [30,43]. Meanwhile, Matthay, MA proposed a new global definition and clinical practice guideline for the European Society of Intensive Care Medicine about acute respiratory distress syndrome to identify the direction of future research, which might be the reason why he received a high citation rate [44,45].

H-index, an author-level scientometric index, was calculated by the number of publications and citations to gauge the significance of a researcher’s work [46]. Although the publications and citations were not high, Hummler, E from the University of Lausanne had the better H-index, who was one of the earliest researchers about functional mutations in human ENaC using transgenic mouse models, and found that α-ENaC-deficient mice tended to early death due to defective neonatal lung liquid clearance [12,47].

**Figure 4:**
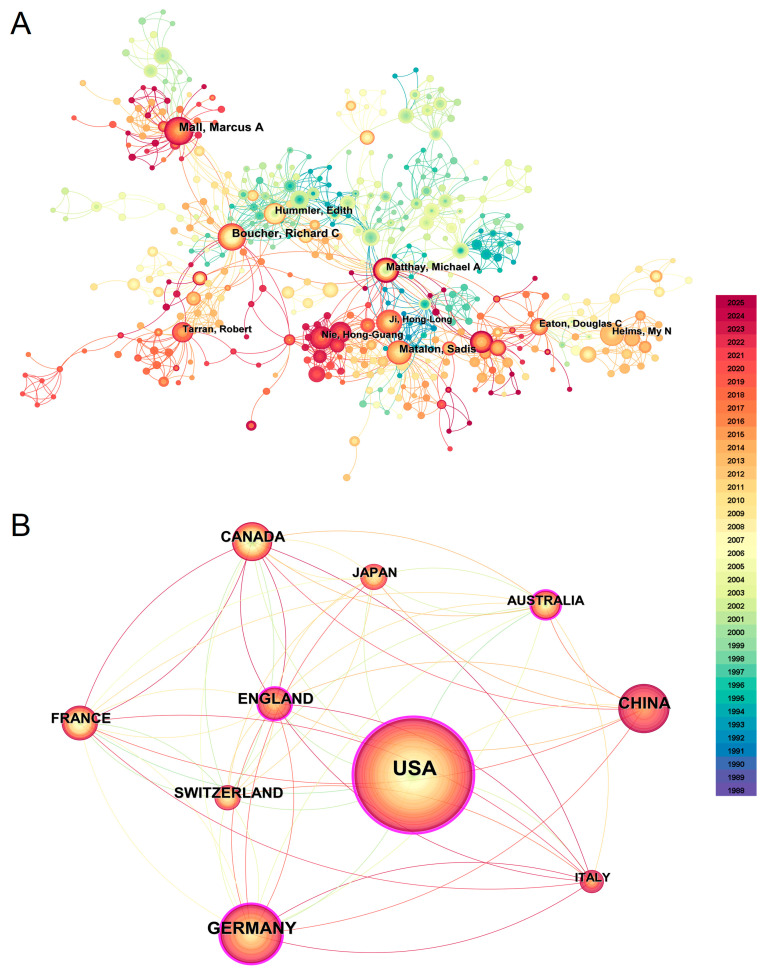
The collaborative network map regarding ENaC in the respiratory system for (**A**) authors and (**B**) countries. The different colors of circles and lines represented the corresponding years, which were listed on the right side of the figure. The circle size represented the number of citations, and the length of lines represented the strength of collaboration.

As shown in Figure 4A, the network map was conducted to reflect the cooperation among all the authors who studied ENaC in the respiratory system. The biggest group in the network included Matthay, MA, Matalon, S, Ji, HL, and Nie, HG, who devoted themselves to the role of ENaC in the proliferative regulation of alveolar epithelial type 2 progenitor cells and blood-gas barrier function [3,48]. The group of Boucher, RC and Hummler, E was followed, and Eaton, DC and Helms, MN were closely behind them. It was noted that Mall, MA, the only author from Germany among the top 10, had loosely connected cooperation, which indicated that his team operated with a relatively independent and self-contained search program, largely centered on cystic fibrosis mouse models [49,50].

Additionally, compared with the authors who had highly cited references, we found that Kellenberger, S was not listed in Table 2 due to just 9 studies published, whereas including two highly cited reviews, implying that the foundation of respiratory ENaC research has been largely consolidated through such seminal literature.

### 4.2. Ranking Publications by Country

The analysis of publications across various nations offered critical insights into the relative priority a country assigns to a particular research domain and its corresponding degree of scholarly influence. A total of 60 countries or regions contributed to publications related to ENaC in the respiratory system, and the information about the top 10 productive countries is shown in Table 3. It was undoubtedly true that the USA ranked top 1 in the number of publications, citations, and H-index, owing to the most productive authors from the USA. Germany, which had the most productive author, Mall, MA, came in second place, and there were other high-output authors, Lucas, R, Duerr, J, and Vadász, I.

The impact of research in a field was not solely determined by the volume of publications, but more importantly by their quality and significance. This was exemplified by the contribution of Switzerland, France, and England to ENaC research, which ranked third, fourth, and fifth in total citations, underscoring their highly influential output despite a modest number of publications, which was consistent with H-index ranking. Considering higher publications and lower citations in China, the studies need to shift from a growth model based primarily on quantity to a more focused strategy of deepening emphasis on quality.

Utilizing Citespace software, we visualized a collaborative network map among the top 10 most productive countries (Figure 4B), which displayed robust research collaboration, with a notable strengthening of ties in recent years. Notably, the network map prominently featured the USA, Germany, and China as major nodes, highlighting their pivotal roles in international partnerships. As we know, Germany acted as a key bridge within the European cluster, while China demonstrated expansive and growing connectivity. Furthermore, the network structure exhibited the USA in North America as the most central hub, facilitating connections between European and Asian research communities, suggesting a growing recognition of the importance of international collaboration in addressing complex scientific challenges and advancing knowledge in the field.

### 4.3. Journals Publishing ENaC Studies

In total, 409 journals published about ENaC in the respiratory system, and 28 journals had more than 10 papers, comprising approximately 6.84% of all the published literature. Table 4 provides the journal influence, documents, and citations of the top 10 most productive journals in the field of respiratory system research related to ENaC, ranked by the number of citations. These journals specialize in physiology and molecular mechanisms, particularly in respiratory and cellular-related categories. Am J Physiol Lung Cell Mol Physiol, which had an impact factor (IF) of 3.5 in 2024, had the highest number of documents (144) and citations (5566), underscoring its central role in disseminating foundational research in this field. While J Biol Chem (IF = 3.9, 2024) and Am J Respir Cell Mol Biol (IF = 5.3, 2024) were ranked as follows. Am J Respir Crit Care Med, in particular, holds the highest IF, reaching 19.4, indicating that its influence was extremely widespread.

Journal Citation Reports (JCR) assigned the journals to quartiles based on IF in their specific subject categories, and evaluated journals based on citation data to reflect the journal’s relative position and influence in its field [51]. Q1, the top 25% journals in a category, represented the most cited and highly cited journals in their discipline. Meanwhile, Q2 was included in 25–50% journals, and regarded as solid and respected outlets with good influence in their field. All of the top 10 most productive journals belonged to Q1 and Q2, with an equal number of journals represented in each category. Moreover, the journals in the respiratory system were classified into Q1, signifying high-quality research with substantial academic impact and broad recognition.

## 5. Emerging Trends

### 5.1. Trend of Research Frontiers

**Figure 5:**
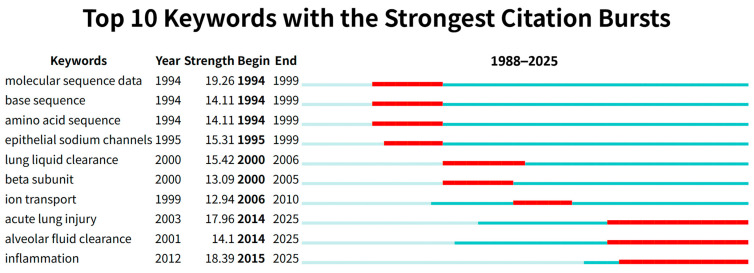
The keywords with the strongest citation bursts of publications about ENaC in the respiratory system. The dark green links represented the year of appearance, whereas the red links represented the year of the strongest citation bursts, from 1988 to 2025 (light green color).

The frontiers of ENaC-related respiratory system were predicted using the strongest citation bursts of publications (Figure 5). Citespace captured the keywords that were identified as research frontiers over time. The top 10 research frontiers of ENaC in the respiratory system were molecular sequence data, base sequence, amino acid sequence, epithelial sodium channels, lung liquid clearance, beta subunit, ion transport, acute lung injury, alveolar fluid clearance, and inflammation. The keyword bursts could be divided into three groups, which were similar to the three phases according to the number of publications. First of all, the molecular sequence data, base sequence, and amino acid sequence broke out before 1999. Then, the regulation and function of ENaC, including lung liquid clearance, beta subunit, and ion transport, were as follows from 2000 to 2010. The last group predicted that the research frontier of ENaC in the respiratory system might witness the role of alveolar fluid clearance and inflammation in acute lung injury [52,53].

### 5.2. Strategic Coordinate Maps

Based on the strategic diagram analysis and thematic evolution mapping of ENaC research from 1988 to 2025, the intellectual structure and developmental trajectory of this field revealed distinct phases of conceptual advancement and thematic consolidation (Table 5). The strategic coordinates illustrated a clear evolution from foundational molecular studies to clinically integrated research themes, with increasing density and centrality indicating maturation in this field.

**Figure 6:**
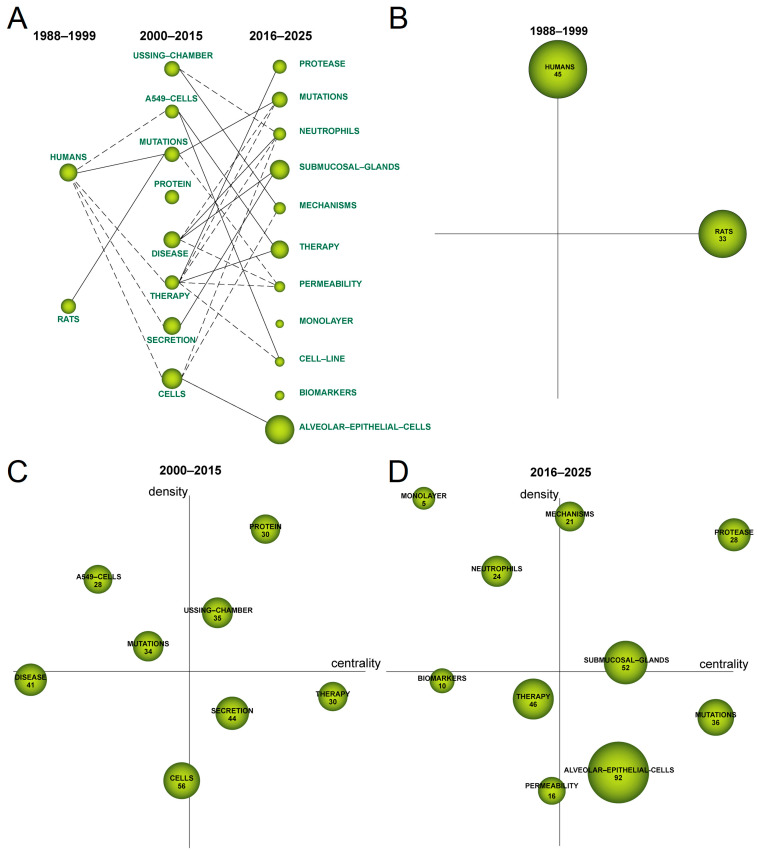
The predicted frontier of ENaC related to the respiratory system. (**A**) Topic evolution chart, the solid line represented conceptual nexus, and the dotted line represented component nexus, strategic diagram of (**B**) 1988–1999, (**C**) 2000–2015, and (**D**) 2016–2025.

At the beginning of this section, in order to obtain an overview of keywords in the development of ENaC in the respiratory system, a three-field plot (Figure 6A) was used. Then the strategic diagrams are articulated in four quadrants (Q). Figure 6B illustrates that the human and rat themes were located in the first and fourth quadrants in the first phase (1988–1999). This theme has both strong external links in various fields and strong internal links among its elements. In the second phase (2000–2015), the core themes were protein and the Ussing chamber, and the themes with potential for development were therapy and secretion, which implied ENaC became an important marker during the treatment of acute lung injury and cystic fibrosis (Figure 6C). The core themes developed protease, mechanisms, and submucosal glands; meanwhile, research using mutations of ENaC in alveolar epithelial cells had extremely high potential (Figure 6D). Moreover, the new themes in the recent phase were the biomarkers of respiratory disease and the role of permeability. Building on the premise that ENaC serves as the first specific biomarker for hypertension, Giosvany’s group successfully optimized a lateral flow immunochromatographic assay to detect the overexpression of this channel in the platelet membranes of hypertensive individuals [54].

## 6. Discussion and Future Perspectives

In the respiratory tract, finely tuned ENaC activity ensures the optimal hydration of airway surface liquid, which is essential for effective ciliary beating and clearance of inhaled particles and pathogens [55,56,57]. Dysregulation of ENaC function is strongly implicated in the pathophysiology of airway diseases. Current bibliometric research faces several challenges, including focusing simply on describing the results produced by bibliometric tools rather than analyzing the data behind them, reliance on a single visualization method for research topics, and a lack of comprehensive analysis of bibliometric results [58]. In this work, we have conducted innovative bibliometric research, analyzing the development process and trend of ENaC in various research fields. The annual numbers of publications, countries, journals, authors, references, and keywords were intuitively displayed.

ENaC is a subject with significant potential for development due to its promising future. A considerable number of papers have been published in highly influential journals. This study performed quantitative and visual analysis of 1634 publications on respiratory ENaC research in the core set of WOS and PubMed databases with the help of CiteSpace (6.4 R1), VOSviewer (v1.6.20), and sciMAT (v1.1.06) software. This study limited literature retrieval to WOS and PubMed, which are the most widely recognized for biomedical bibliometric analysis. Using the same retrieval strategy, we have also tried other databases such as Scopus and Dimensions, which could offer 1331 publications. Besides those from the WOS and PubMed databases, there were 93 unique publications that might have been missed in the present study. Our future research may expand into these databases for identifying additional literature, and we will strive to explore cross-database comparisons for even broader coverage. The USA is the leading country in productive authors and institutions in this field. Boucher, RC, Mall, MA, Matthay, MA, Matalon, S, Hummler, E, Eaton, DC, Tarran, R, Ji, HL, Helms, MN, Nie, HG are prominent scientific leaders in this research field. Collaboration and communication among countries and authors are strongly recommended [59]. Most studies in the past years have focused on the regulation and function of ENaC, as well as the therapy and pathophysiology of respiratory disease. In this study, we found research orientation changed to solve pathogenesis such as inflammation, permeability and alveolar fluid clearance. Recent advances illuminated the complex regulation of ENaC by mechanical forces, inflammatory mediators, and proteases derived from both host and microbial sources [60,61]. miRNA-130b from bone marrow mesenchymal stem cells can enhance ENaC expression by targeting PTEN and activating the PI3K/AKT pathway, which may provide a promising new direction for therapeutic strategy in acute lung injury [62]. Furthermore, emerging studies highlight ENaC’s involvement in innate immunity, epithelial barrier integrity, and crosstalk with other ion transport pathways such as CFTR and calcium-activated chloride channels [63,64,65]. These findings suggest that ENaC is not only a mediator of fluid balance, but also a broader modulator of airway physiology and disease.

Clinical studies are warranted to test whether ENaC expression or activity may become a promising therapeutic target in respiratory diseases. Pharmacological approaches ranging from direct channel blockers, such as NVP-QBE170 and AZD5634, to novel modulators that fine-tune channel activity are being explored in preclinical and clinical settings [16,66]. However, clinical trials about targeting ENaC have been impeded by the difficulty of overcoming heterogeneous mucus obstruction. Although still needing to rely on more experimental and clinical data, the bibliometric analysis in this study highlighted that the diverse roles of ENaC became a focal point of current research, proposing future strategies for managing respiratory diseases, characterized by fluid imbalance and impaired mucociliary clearance.

Future studies should explore more deeply the inflammatory responses and alveolar fluid clearance mechanisms involved in the regulation of the respiratory system by ENaC, and clinical studies should be strengthened to verify the biomarker of ENaC in respiratory diseases. As the unique subunit in humans, the regulation of δ-ENaC may contribute to precision medicine, because the two known isoforms of δ-ENaC subunit can build amiloride-sensitive sodium channels with distinct pharmacological properties [67]. Organizations can refer to this article as a reference when deciding whether to provide repeatable supporting funding to a given research team [68]. In the future, institutions should integrate and complement their research fields on ENaC in the respiratory system.

Nevertheless, it is important to acknowledge several limitations of this study. Firstly, the results were processed with certain algorithms, which could lead to bias in some of the results. Then, only English articles were included from the database and analysis, potentially leading to a source bias. Additionally, some high-quality studies from the data may be omitted due to insufficient citation frequency, and the delayed citation impact often associated with recently published work, given that bibliometric analysis heavily depends on citation counts [69]. Despite these limitations, bibliometric analysis continues to offer valuable insights, allowing for a broad and efficient overview of the research landscape within a given field.

## 7. Conclusions

This bibliometric study defines the overall prospects and may provide reference or enlightenment for scientific workers engaged in the research of respiratory ENaC research. The bibliometric mapping provides a data-based approach to identify the emerging frontier research field of crosstalk between the immune and ENaC.

## Figures and Tables

**Table 1:** The top 10 cited publications conducted to research ENaC in the respiratory system.

Rank	Title	Journal	Total Citations	Publication Year	First Author	Document Type
1	Rescue of CF airway epithelial cell function in vitro by a CFTR potentiator, VX-770	Proc Nat Acad Sci USA	1011	2009	Van Goor, F	Article
2	Epithelial sodium channel/degenerin family of ion channels: a variety of functions for a shared structure	Physiol Rev	853	2002	Kellenberger, S	Review
3	Increased airway epithelial Na^+^ absorption produces cystic fibrosis-like lung disease in mice	Nat Med	738	2004	Mall, MA	Article
4	Evidence for airway surface dehydration as the initiating event in CF airway disease	J Intern Med	288	2007	Boucher, RC	Review
5	Epithelial Na^+^ channels are fully activated by furin- and prostasin-dependent release of an inhibitory peptide from the gamma-subunit	J Biol Chem	276	2007	Bruns, JB	Article
6	Structure and regulation of amiloride-sensitive sodium channels	Annu Rev Physiol	264	2000	Álvarez de la Rosa, D	Review
7	Regulation and repair of the alveolar-capillary barrier in acute lung injury	Annu Rev Physiol	262	2013	Bhattacharya, J	Review
8	Elevated Plasmin(ogen) as a Common Risk Factor for COVID-19 Susceptibility	Physiol Rev	258	2020	Ji, HL	Review
9	Acid-sensing ion channels in sensory perception	J Biol Chem	250	2007	Lingueglia, E	Review
10	International Union of Basic and Clinical Pharmacology. XCI. structure, function, and pharmacology of acid-sensing ion channels and the epithelial Na^+^ channel	Pharmacol Rev	224	2015	Kellenberger, S	Review

**Table 2:** The top 10 most productive authors ranked by citations.

Rank	Author	Institution	Country	Publications	Citations	H-Index
1	Boucher, RC	University of North Carolina	USA	60	3936	31
2	Mall, MA	Charité-Universitätsmedizin Berlin	Germany	66	3636	32
3	Matthay, MA	University of California, San Francisco	USA	47	2708	23
4	Matalon, S	University of Alabama, Birmingham	USA	56	2213	29
5	Hummler, E	University of Lausanne	Switzerland	40	1966	25
6	Eaton, DC	Emory University	USA	46	1693	23
7	Tarran, R	University of Kansas	USA	35	1227	20
8	Ji, HL	Loyola University Chicago	USA	32	1053	17
9	Helms, MN	University of Utah	USA	43	915	18
10	Nie, HG	China Medical University	China	38	467	13

**Table 3:** The top 10 most productive countries ranked by citation.

Rank	Country	Publication	Total Citation	Average Citation	H-Index
1	USA	740	29,751	40	85
2	Germany	238	7534	32	50
3	Switzerland	102	5554	54	39
4	France	87	4118	47	37
5	England	111	3471	31	36
6	China	168	3078	18	29
7	Canada	97	2778	29	33
8	Australia	55	1929	35	27
9	Japan	60	1322	22	19
10	Italy	43	1208	28	20

**Table 4:** The top 10 most productive journals.

Rank	Journal	IF (2024)	JCR (2024)	Document	Citations
1	Am J Physiol Lung Cell Mol Physiol	3.5	Q1	144	5566
2	J Biol Chem	3.9	Q2	60	3730
3	Am J Respir Cell Mol Biol	5.3	Q1	51	1963
4	Am J Respir Crit Care Med	19.4	Q1	47	1358
5	Plos one	2.6	Q2	25	767
6	Pflugers Arch	2.8	Q2	29	751
7	Am J Physiol Cell Physiol	4.7	Q2	25	660
8	FASEB J	4.2	Q1	51	242
9	Int J Mol Sci	4.9	Q1	21	157
10	Pediatr Pulmonol	2.3	Q2	41	92

**Table 5:** Key information of the strategic coordinate chart.

Phase	Q1	Q2	Q3	Q4
1988–1999	Humans			Rats
2000–2015	Protein	A549 cells	Disease	Secretion
	Ussing chamber	Mutations	Cells	Therapy
2016–2025	Protease	Monolayer	Biomarkers	Alveolar epithelial cells
	Mechanisms	Neutrophils	Therapy	Mutations
	Submucosal glands		Permeability	

## Data Availability

Data will be made available on request.
